# Minimal Residual Disease Surveillance in Chronic Lymphocytic Leukemia by Fluorescence-Activated Cell Sorting

**DOI:** 10.5041/RMMJ.10161

**Published:** 2014-10-29

**Authors:** Shimrit Ringelstein-Harlev, Riva Fineman

**Affiliations:** 1 Department of Hematology and Bone Marrow Transplantation, Rambam Health Care Campus, Haifa, Israel; 2 Bruce Rappaport Faculty of Medicine, Technion, Israel Institute of Technology, Haifa, Israel

**Keywords:** ASO-PCR, chronic lymphocytic leukemia, flow cytometry, minimal residual disease, prognosis, survival

## Abstract

Achievement of complete response (CR) to therapy in chronic lymphocytic leukemia (CLL) has become a feasible goal, directly correlating with prolonged survival. It has been established that the classic definition of CR actually encompasses a variety of disease loads, and more sensitive multiparameter flow cytometry and polymerase chain reaction methods can detect the disease burden with a much higher sensitivity. Detection of malignant cells with a sensitivity of 1 tumor cell in 10,000 cells (10^–4^), using the abovementioned sophisticated techniques, is the current cutoff for minimal residual disease (MRD). Tumor burdens lower than 10^–4^ are defined as MRD-negative. Several studies in CLL have determined the achievement of MRD negativity as an independent favorable prognostic factor, leading to prolonged disease-free and overall survival, regardless of the treatment protocol or the presence of other pre-existing prognostic indicators. Minimal residual disease evaluation using flow cytometry is a sensitive and applicable approach which is expected to become an integral part of future prospective trials in CLL designed to assess the role of MRD surveillance in treatment tailoring.

## INTRODUCTION

Chronic lymphocytic leukemia (CLL), a CD5^+^ mature B-cell malignancy, has long been considered an incurable disease, and its treatment was focused on symptom control rather than on survival prolongation. This situation has changed dramatically over the past two decades as new effective treatment strategies have emerged, leading to achievement of complete response (CR) in a considerable number of patients. It is now established that achievement of CR according to the CLL International Workshop (IW-CLL) guidelines^1^ is associated with prolonged disease-free survival. In agreement with this, the analysis of data from the phase II FCR (rituximab addition to fludarabine and cyclophosphamide) study conducted at the MD Anderson Cancer Center showed that CR was the most important determinant of long-term survival. In this study, inferior survival and decreased CR rates were associated with adverse pre-treatment factors; however, patients who managed to achieve CR demonstrated durable remissions, no matter whether they presented with adverse prognostic factors.^2^

Nevertheless, CR defined by clinical findings, blood count, and bone marrow cellularity is not equivalent to disease eradication, and the classic definition of CR actually includes patients harboring 10^1^–10^10^ neoplastic cells. If indeed achievement of CR forecasts freedom from disease, it is conceivable that the deeper the CR, the greater the effect on disease suppression and perhaps even on overall survival and cure. New combination treatments which incorporate biologic agents and monoclonal antibodies into the treatment regimens induce deeper responses, leading to a higher incidence of CR; however, more sensitive techniques for residual disease detection would probably show that the CR group actually encompasses a vast variety of responses. Therefore, the incorporation of sensitive techniques into disease status evaluation in histological negative malignancies allows a more precise segregation of treatment responses, which is vital for clinical decision-making and prognostic purposes.

## MINIMAL RESIDUAL DISEASE IN HEMATOLOGIC MALIGNANCIES

Assessment of minimal residual disease (MRD) as a measurement of tumor load in hematologic malignancy is becoming a tool for monitoring the depth of disease response, predicting early relapse, and tailoring treatment. Monitoring MRD as a tool for detecting disease presence in patients without histological evidence of the tumor has been incorporated into studies in acute lymphoblastic leukemia (ALL), acute promyelocytic leukemia (APL), as well as mantle cell lymphoma (MCL).^3^–^5^ Positivity and negativity of MRD are defined by the threshold for detection of malignant cells among total nucleated cells, estimated with modern techniques to be around 1:100,000. In ALL, MRD measurement at different time points throughout and following treatment (MRD surveillance) was found to be the most powerful predictor for relapse^3^ and was later included into studies augmenting treatment according to MRD positivity in standard- and intermediate-risk patients.^6^ In MCL, Andersen et al. have shown that pre-emptive rituximab in patients becoming MRD-positive following MRD negativity returned them to a negative status, prolonging molecular and clinical freedom from disease.^4^

Minimal residual disease in B-cell malignancies has been monitored in clinical studies using two major methods. The first is fluorescence-activated cell sorting (FACS), also referred to as “flow cytometry” (FLC), which detects cells harboring a specific phenotype and depicts their number among the normal cell population, and the second is allele-specific oligonucleotide polymerase chain reaction (ASO-PCR) which sequences and amplifies patient-specific DNA in the immunoglobulin heavy-chain variable region (IgHV). Of note, FLC is usually used for detecting CLL cells at diagnosis and following treatment, but routine laboratory methods cannot detect a low tumor burden and are thus not valid for MRD analysis. Hence, a distinction must be made between MRD evaluation and routine disease detection by FLC.

Implementation of MRD assessment into clinical practice lags behind due to the lack of standardized detection methods and absence of adequate laboratory facilities in some centers. Moreover, evidence-based guidelines for decision-making based on MRD are still unavailable. Another issue related to the applicability of MRD assessment in routine practice is that certain malignancies show vast heterogeneity of markers best defining them; hence, designing a standard routine method that will fit everyone is difficult.

## MINIMAL RESIDUAL DISEASE IN CLL

Minimal residual disease assessment in CLL began two decades ago in studies utilizing poorly specific and insensitive two-color FLC methods, referring to all CD19^+^CD5^+^ light-chain restricted cells as CLL. Back in 1992, Robertson et al.^7^ demonstrated that even when MRD negativity is defined by basic two-color FLC, CR patients can be divided into two groups, with differences in progression-free survival (PFS) between MRD-positive versus negative patients (19 months versus over 30 months, respectively). O’Brien et al.^8^ also showed a small but significant prolongation of PFS in MRD-negative CR patients after fludarabine and cyclophosphamide treatment in a small group of 36 patients analyzed by the same insensitive FLC technique.

Later studies began using more complex multi-color multiparameter FLC as well as PCR to assess MRD as a secondary end-point, and examined a variety of treatment regimens, coming up with results in line with those presented above. These findings added solid evidence for the role of MRD surveillance in CLL during first-line as well as advanced-line treatments.

### Minimal Residual Disease Negativity in First-Line Therapies

In a study by Bosch et al.,^9^ 69 patients were treated with the fludarabine/cyclophosphamide/mitoxantrone (FCM) protocol as front-line therapy. Minimal residual disease status was assessed by multiparameter (four-color) FLC and ASO-PCR. Twenty-six percent of patients achieved MRD-negative CR and had a lower probability of progression at 2 years (9% for MRD-negative patients versus 20% for MRD-positive patients). Moreover, MRD-negative CR also led to improved overall survival (OS) compared to any inferior response.

Hillmen et al.^10^ reported similar results by analyzing 297 previously untreated patients who received alemtuzumab versus chlorambucil. In this study, four-color FLC demonstrated an MRD-negative status in 26% of CR patients, and, in consensus with the study by Bosch et al., MRD-negative CR patients demonstrated a significantly improved PFS compared to those with MRD-positive CR.

The most substantial evidence for the benefit of achieving MRD negativity in first-line therapies comes from the German CLL Study Group (GCLLSG) trial designed, among other things, to compare MRD status in two different arms using four-color FLC 2 months after treatment.^11^ In this study, 817 untreated patients were randomized to either fludarabine and cyclophosphamide (FC) or FCR treatment protocols. Complete response rates were significantly higher with FCR (52%) than with FC (27%), as was the MRD negativity status in the bone marrow (47.6% and 27.3%, respectively). Ten-year follow-up of patients in the FCR arm displays what seems to be a plateau in survival curves, arising mostly from the low MRD patient group, suggesting that some of these patients may have been cured. Of interest, MRD levels were found to be of higher prognostic value than the treatment regimen itself, cytogenetics (excluding the 17p deletion), pre-therapeutic white blood cell count, β2-microglobulin, and IgHV mutational status.

A retrospective analysis of over 200 patients treated with a variety of first-line therapies conducted by Santacruz et al.^12^ supports the above findings, demonstrating clearly that MRD negativity was a consistent predictor of both treatment-free survival and OS, with an almost doubled treatment-free survival interval for patients achieving MRD-negative CRs (76 versus 40 months). This analysis also highlights that the advantages of MRD negativity achievement are not confined to one specific treatment protocol.

### Minimal Residual Disease Negativity in Advanced-Line Therapies

The issue of MRD negativity has also been addressed in advanced-line treatments. In the study by Moreton et al.,^13^ 91 patients refractory to purine analogues were treated for a total of 9 weeks with alemtuzumab, and assessed for bone marrow MRD by four-color FLC. In this study treatment-free survival as well as median survival were significantly longer in MRD-negative patients compared with those achieving an MRD-positive CR, partial response, or no response. Median treatment-free survival for MRD-negative patients had not been reached at 60 months, versus 20 months for those with MRD-positive CRs.

In a similar vein, among 37 patients with resistant or relapsed CLL treated with FCM in the study by the Spanish GELCAB (Grup per l’Estudi dels Limfomes a Catalunya i Balears),^14^ median duration of PFS and overall response was longer for patients achieving MRD-negative versus MRD-positive CR.

### Minimal Residual Disease Negativity in the Post-allogeneic Stem Cell Transplant Setting

Minimal residual disease has also been evaluated in the post-allogeneic stem cell transplant (alloSCT) setting. Post-alloSCT follow-up presents challenges in decision-making due to the fact that while physicians have a potential therapeutic tool that can be constantly manipulated for disease control, studies have not determined the exact scenarios requiring intervention, or the best intervention method. A study by the German CLL Group examined the MRD status at several time points after alloSCT and demonstrated enhanced event-free survival in MRD-negative patients at 12 months.^15^ This finding emphasizes not only the importance of MRD negativity per se, but also the significance of performing MRD analysis at the 12-month time point in alloSCT patients. Another small retrospective analysis^16^ also addressed this issue demonstrating similar data regarding the correlation between MRD negativity and disease-free survival, and suggesting a potential role for sequential MRD monitoring, since the dynamics of MRD level proved to be a relevant prognostic factor.

Importantly, these two studies report an association between MRD surveillance per se and improved event-free survival, suggesting that once the treating physician was equipped with the knowledge of MRD status, treatment modifications were made leading to improved survival. Optional treatment modifications applied in MRD-positive patients may include the use of donor lymphocyte infusions or reduction of immunosuppressive drug dose. This observation emphasizes the need for trials specifically addressing the question of treatment tailoring according to MRD dynamics after transplantation.

### Can MRD Status Guide Risk Adapted Therapy in CLL?

The available data imply that MRD negativity is a potent landmark on the way to improved survival, raising questions about its potential applicability for treatment de-escalation and toxicity reduction in patients having reached MRD negativity early in the course of therapy. Two prospective studies addressed this issue suggesting that MRD negativity can guide decisions regarding therapy duration and intensity. In the study by Strati et al.,^17^ MRD negativity in bone marrow samples after three cycles of FCR resulted in the same PFS and OS as those found in patients with no detectable MRD after six courses of FCR. Data analysis of the GCLLSG CLL8 study reveals a similar picture, showing that PFS was similar in patients who had already achieved low MRD levels after three treatment cycles compared with those who required the full treatment (six cycles) to attain this status.^11^

These two studies show that MRD negativity is an independent prognostic factor at any time point, suggesting that once reached it may provide sufficient relapse risk “protection” which is not enhanced with treatment continuation.

If so, strict adherence to the full-length treatment protocols might prove to be unwarranted, and shortening or de-escalation of therapy could reduce toxicity in patients achieving early and deep responses. This hypothesis needs to be proven in a randomized trial, but currently available data are nevertheless thought-provoking.

## STANDARDIZATION OF MRD ANALYSIS TECHNIQUES

One of the limitations in the interpretation of presented data is related to the fact that the studies have employed different methods of MRD measurement, which complicates the comparison. However, at least three studies^10^,^11^,^16^ utilized a protocol that was published by the European Research Initiative on CLL (ERIC), which proposed an international standardized approach (ISA) and identified three four-color antibody combinations (CD5/CD19 with CD20/CD38; CD81/CD22; and CD79b/CD43) for the detection of MRD by FLC in CLL,^18^ providing recognition of residual leukemic cells at a level of 10^−4^ (or 1 malignant cell in 10,000 normal cells),^19^,^20^ which is now acknowledged as the standard level for MRD negativity. According to these guidelines, MRD should be detected in at least two of the three fluorescent antibody combinations, assessed after acquiring at least 200,000 events per tube, which can be done only when staining at least 1×10^6^ cells per test tube. Conceptually, cells are distinguished by their differential co-expression of CD19 and CD5 together with the absence or low expression of CD22, CD79b, and CD81, and over-expression of CD43. Specific gating strategies have been established for sample analysis according to this standardization. The following test tubes are required: (1) CD20 CD38 CD19 CD5; (2) CD81 CD22 CD19 CD5; (3) CD43 CD79b CD19 CD5.

Two additional tubes are recommended: (1) a screening tube containing the sIgλ SIgk CD19 CD5 combination, which can detect above 10^−2^ cells with a 100% positive predictive value, thus obviating the need to proceed with more extensive tests if clearly demonstrating residual CLL cells, and (2) an antibody combination that is becoming increasingly important, and includes CD45 CD14 CD19 CD3, for the removal of contaminating double-stained non-B-cells and for setting the detection threshold.

For MRD-positive samples, the consensus for reporting the level of MRD in CLL is the percentage of CLL cells among all leukocytes after indicating the limit of detection. For MRD-negative samples, the report should specify that CLL percentage is under the indicated level of detection. If the limit of detection is above 10^−4^, then a comment should be made indicating that the sample is inadequate, and an explanation is to be provided.

## SUGGESTED IMPROVEMENTS FOR THE ISA PANEL

Flow cytometry is an accessible and inter-laboratory reproducible method, and, as such, it yields results that can be incorporated into real-time decisions.

Following the ISA publication, other groups have proposed alternative combinations with the intent to optimize cost and complexity of the ISA panel, or even improve its sensitivity.

The same group that issued the 2007 ISA approach conducted further studies under the auspices of the European Research Initiative in CLL, and proposed an alternative six-color FLC assay^21^ that incorporated CD3 into tubes co-stained for CD19/CD5/CD20/CD79b/CD38, demonstrating the importance of removing the group of contaminating CD3^+^CD19^+^ that had otherwise been defined as CLL and lowered the positive predictive value of MRD positivity. This method also reduced time and complexity of the analysis and allowed for analysis of cases with very hypocellular marrow or lymphopenia due to reduction in the number of events required for analysis. Very good concordance with the four-color assay was observed for detection in the range of 10^−4^–10^−5^.

Raponi et al.^22^ examined an even more complex eight-color panel, including CD81/CD38/CD20/CD43/CD5/CD45/CD19/CD3, in a very limited number of patients and compared it to the ERIC consensus panel with a good overlap of MRD results.

A study by Sartor et al.^23^ incorporated a 10-color FLC combination, based on the ISA combination, into a trial examining lenalidomide maintenance in MRD-positive patients. The authors speculated that the number of cells needed to be acquired could be decreased, which is, as mentioned, an advantage in patients who are lymphopenic post-therapy, and more easily exclude contaminating T-cells by placing all antibodies in the same tube. Eighty samples analyzed by both the ISA and the proposed 10-color assay showed a strong correlation between the methods, both capable of reaching sensitivity of 3×10^−5^, but the 10-color assay could eliminate contaminating CD3^+^CD19^+^ cells more efficiently, improving the positive predictive value of the test, and lowering the number of leukocytes required for analysis from 1×10^7^ in the ISA to 2×10^6^.

## COMPARISON OF METHODS FOR MRD DETECTION

As previously mentioned, FLC is not the only method employed in MRD studies in CLL. While ASO-PCR is generally considered a more sensitive method,^19^ detecting 10^−4^–10^−6^ disease cells, it requires patient-specific reagents and is laborious and expensive. Its results can also be obscured by low quality and quantity of DNA. According to this method, DNA is amplified with primers specific to the immunoglobulin gene rearrangement present in the neoplastic cell following individual patient sequencing of the variable domain region of the immunoglobulin. This is indeed a very sensitive technique; however, only real-time PCR can provide a quantitative answer regarding the number of malignant cells, a prerequisite for precise MRD evaluation. Therefore, a combination of real-time PCR with ASO-immunoglobulin heavy-chain variable region (IgHV) primers is required for sensitive quantitative measurement of PCR copy numbers.

Naturally, efforts have been made to compare both methods of MRD surveillance in an attempt to select the best sensitive but yet applicable method. The study by Raponi et al.^22^ conducted a comparative analysis of FLC and real-time ASO-PCR for MRD monitoring in CLL. This was done by monitoring blood and bone marrow samples from 98 patients, establishing a consensus of 82% between the two methods. With ASO-PCR serving as a reference, the sensitivity and specificity of MRD assessment by FLC were 96.5% and 77.2%, respectively. Positive predictive value was 57.1%, and negative predictive value was 98.6%.

Bottcher et al.^20^ also conducted a comparative study of molecular and FLC techniques following autologous and allogeneic SCT. This comparison showed FLC to be less sensitive than real-time quantitative (RQ)-ASO-PCR, with 15% of patients found negative by FLC actually being positive by PCR. However, two facts lead to the conclusion that both methods are equally suitable to measure MRD in CLL patients: (1) within the common sensitivity range, there is good concordance between the methods, and (2) MRD FLC was consistently capable to detect lower MRD levels than RQ-ASO-PCR.

Finally, Rawstron et al.^18^ also conducted a comparison between four-color FLC and RQ-ASO-PCR and found excellent concordant results at the 10^−4^ disease level. Notably, in this study, as opposed to the investigation by Bottcher et al.,^20^ when considering the detection of CLL cells at lower levels, the PCR approach had higher sensitivity.

Another question encountered in practice is whether peripheral blood (PB) examination suffices for MRD analysis. The study conducted by Rawstron et al.^24^ according to ERIC ISA guidelines demonstrates that PB assessment is as good as that of the bone marrow, with the exception of the first few months after treatment when residual disease present in the marrow may not be detected in PB, particularly in patients treated with monoclonal antibodies targeting CLL.

Raponi et al.^22^ also addressed this question and found an approximately 92% concordance between results in bone marrow and PB; however, among the discordant 8% almost all were positive in bone marrow and negative in PB, implying that true MRD negativity cannot be unequivocally determined using PB only.

## SUMMARY AND A LOOK TO THE FUTURE

The available data provide strong evidence for the incorporation of MRD assessment into two aspects of CLL patient care. It may serve as an independent individualized prognostic indicator predicting PFS and OS in patients with CLL, regardless of the type of treatment, and it may guide treatment-related decision-making.

Available data support the determination of MRD levels using the ERIC consensus four-color FLC in PB, since its high sensitivity and negative predictive value compared to RQ-ASO-PCR, as well as good concordance with bone marrow findings, reassure that positive cases are rarely missed. Flow cytometry assays utilizing more than four colors in each study tube may replace the ISA in the near future. Certain scenarios, such as MRD assessment earlier than 3 months after treatment, or analysis of extremely low numbers of cells, may warrant bone marrow examination and the use of RQ-ASO-PCR; however, these scenarios are limited.

Further studies are needed to resolve issues regarding the optimal time points for MRD evaluation following different treatment protocols and the value of MRD surveillance in the era of new biological agents. The issue of appropriate clinical decisions based on MRD levels is unresolved. The assumption that MRD level could guide chemoimmunotherapy tailoring or pre-emptive immune manipulations in alloSCT patients remains to be investigated. Notably, in the alloSCT setting, interventions based on MRD data are already a working theory, although not yet evidence-based.

While the information about prospective studies intended to guide real-time treatment decisions according to MRD levels is extremely limited, at least one such trial is underway, examining lenalidomide maintenance therapy for MRD-positive patients concluding chemo-immunotherapy (the study by the Australian Lymphoma and Leukemia Group). The CLL International Workshop has recently recommended investigating the significance of MRD in clinical studies as a priority; so, the proportion of such studies among the long list of clinical trials in CLL is expected to rise in the upcoming years. Studies of MRD-tailored therapy could establish the role of MRD monitoring as a tool for extending survival by treatment escalation in MRD-positive patients, on the one hand, and as a tool for minimizing toxicity by treatment de-escalation in MRD-negative patients, on the other hand.

## Abbreviations:

ASO-PCR: allele-specific oligonucleotide polymerase chain reaction;
CLL: chronic lymphocytic leukemia;
CR: complete response;
ERIC: European Research Initiative in CLL;
FC: fludarabine and cyclophosphamide;
FCM: fludarabine/cyclophosphamide/mitoxantrone;
FLC: flow cytometry;
IgHV: immunoglobulin heavy-chain variable region;
ISA: international standardized approach;
MRD: minimal residual disease;
OS: overall survival;
PB: peripheral blood;
PFS: progression-free survival;
RQ: real-time quantitative.
